# Clinical Practice Guidelines For the Management of Hepatocellular Carcinoma: A Systematic Review

**DOI:** 10.1007/s12029-023-00961-0

**Published:** 2023-07-22

**Authors:** Ishith Seth, Adrian Siu, Lyndel Hewitt, Ulvi Budak, Beshoy Farah, Mouhannad Jaber

**Affiliations:** 1 Illawarra Shoalhaven Local Health District, Wollongong Hospital, Wollongong, NSW 2500 Australia; 2 Illawarra Health and Medical Research Institute, Wollongong, NSW 2522 Australia; 3 Faculty of Science, Medicine and Health, University of Wollongong, Wollongong, NSW 2522 Australia; 4 School of Medicine, Graduate Medicine, University of Wollongong, Wollongong, NSW 2522 Australia; 5 Faculty of Medicine and Health Sciences, Monash University, Victoria, 3004 Australia

**Keywords:** Hepatocellular carcinoma, HCC, Cancer, Liver, Clinical guidelines, Guidelines

## Abstract

**Background:**

Hepatocellular carcinoma (HCC) is a leading cause of cancer-related deaths globally, including Australia. The absence of a consensus clinical practice guideline (CPG) specific to HCC management poses challenges in reducing morbidity, mortality, and improving patient recovery. This systematic review aims to evaluate the existing evidence and assess the potential of published guidelines, including those with an international scope, to provide guidance for healthcare professionals in Australia.

**Methods:**

Electronic search of MEDLINE, Embase, Cochrane Library, Google Scholar, and PubMed was conducted. Peer-reviewed English language articles from 2005 to June 2022 were included if they described management of HCC as part of an evidence-based overall management plan or CPG. The quality of the included CPGs was assessed by the Appraisal of Guidelines for Research and Evaluation II (AGREE II) tool.

**Results:**

Twenty-one articles from 16 regions throughout the world were included in this review. All included guidelines (*n* = 21, 100%) recommended evaluating cirrhosis, hepatitis B, and hepatitis C as potential risk factors of HCC. Obesity and non-alcoholic fatty liver disease were recommended by 19 CPGs (91%) as risk factor for HCC. Fourteen guidelines (67%) endorsed using the BCLC staging system. Eighteen guidelines (86%) recommended a multidisciplinary approach for the management of HCC. Eighteen guidelines (86%) advised that surveillance using ultrasound should be implemented in all cirrhotic patients every 6 months regardless of the cause of cirrhosis. AGREE II mean overall assessment score was 90% indicating that all guidelines included were highly recommended in majority of domains.

**Conclusions:**

The included CPGs provided a comprehensive approach, emphasizing the evaluation of risk factors, utilization of the BCLC staging system, and the importance of a multidisciplinary approach. Regular surveillance using ultrasound for cirrhotic patients was widely recommended. An understanding of contemporary international CPGs can prioritize aspects of the management of HCC to assist healthcare professionals to develop a national guideline to enable standardized, comprehensive, and evidence-based care for patients with HCC.

## Introduction

Hepatocellular carcinoma (HCC) is the sixth leading malignancy and the third leading cause of cancer deaths globally [1]. This disease constitutes approximately 90% of primary liver cancers and represents a significant global health issue. Internationally, there were greater than 800,000 new cases of HCC in 2018, with 781,631 deaths [2]. Additionally, incidence of HCC in developing countries was two to three times higher than in developed countries. This is reflected in the age-adjusted incidence rates of 20–28 cases per 105 males in Middle Africa and Eastern Asia, compared to 1–3 cases per 105 in North American and Northern Europe [3]. Of note, the prevalence and mortality rates of HCC in men are two to three times higher than women in most countries [4].

Hepatocellular carcinoma exhibits considerable variation in its etiology across different regions of the world. The diverse etiological factors associated with HCC are crucial to consider when designing effective screening practices and evaluating their cost-effectiveness. In various high-income countries, chronic hepatitis B and C infections, as well as alcohol-related liver disease, remain prominent etiological factors for HCC [5]. Conversely, in regions with a high prevalence of chronic hepatitis B, such as parts of Asia and sub-Saharan Africa, viral infection plays a more significant role [6]. Additionally, the emergence of non-alcoholic fatty liver disease (NAFLD) as a leading cause of HCC in Western countries further highlights the evolving global landscape. Other contributing factors, such as aflatoxin exposure in certain regions of Africa and Asia, as well as hereditary conditions like hemochromatosis, Wilson’s disease, and alpha-1 antitrypsin deficiency, add to the complexity of HCC etiology worldwide. The single main risk factor for the occurrence of HCC is cirrhosis of any underlying etiology [7]. By understanding these global variations in etiology, we can develop tailored screening approaches that account for the specific risk factors prevalent in each region, ultimately enhancing the effectiveness and cost-effectiveness of HCC screening practices on a global scale.

Over the past 2 decades, several clinical practice guidelines (CPGs) have documented the clinical management of HCC. However, the overall outcomes of HCC are still far from satisfactory. There is no consensus of world CPGs for HCC. We have conducted a broad search of the literature to provide a comprehensive summary of HCC management. The aim was to determine the management of HCC, including risk factors, staging, diagnosis, treatment options, prevention, surveillance, and follow-up. The included CPGs offer up-to-date advice for the clinical management of patients with HCC and critically reviews all relevant data leading to the conclusions of this article.

## Methods

This systematic review was registered on PROSPERO, the International Prospective Register of Systematic Reviews (http://www.crd.york.ac.uk/prospero, registration no. CRD42021271122). This study was conducted according to the Preferred Reporting Items for Systematic Reviews and Meta-Analyses (PRISMA) statement [8].

### Search Strategy

MEDLINE, Embase, PubMed, Google Scholar, and Cochrane Library databases were searched for relevant articles from infinity to 31^st^ June 2022. The search was limited to titles and abstracts containing ‘hepatocellular carcinoma*’ OR ‘HCC’ or ‘liver cancer’ AND ‘clinical practice guideline*’ OR ‘guideline’ OR ‘practice guideline’ OR ‘consensus’ OR ‘position statement*.’ Endnote X9 (https://endnote.com) was utilized to remove duplicates and to manually screen the search output. Two researchers (IS and AS) independently reviewed the titles and abstracts to determine their eligibility for inclusion. Full-text papers were retrieved and studied to confirm eligibility. Differences between reviewers were discussed with the research team to define an inclusion agreement. The references of the full-text articles were also evaluated for relevant studies.

### Inclusion and Exclusion Criteria

This review included studies of HCC patients at any initial surveillance, diagnostic or peri-operative stage (pre-operative, intra-operative, and post-operative) using a CPG, or evidence-based management plan. The articles were required to be peer-reviewed English language articles, from 2005 to 2022, containing a description of the management of HCC that is part of an overall evidence-based management plan, guideline, or CPG. Studies regarding the management plan for other types of cancer or other medical conditions were excluded. Book chapters, dissertations, case reports/series, and conference abstracts were also excluded.

### Included Outcomes

#### Risk Factors

Many risk factors can predispose to HCC. These include cirrhosis, hepatitis B virus (HBV), hepatitis C virus (HCV), alcohol, aflatoxin, obesity, non-alcoholic fatty liver disease (NAFLD), hereditary hemochromatosis, Wilson disease, type 1 glycogen storage disease, metabolic disorders, and NASH.

#### Staging

The staging of HCC is vital to the prediction of survival and selection of an appropriate treatment strategy. The prognosis of HCC is predominantly affected by tumor factors, the patient’s general state, and the function of the liver. Several staging systems are used in clinical practice—the Barcelona Clinic Liver Cancer (BCLC) staging (which includes five stages which assess the number and size of tumor from stage 0 indicating very early stage disease and stage D indicating severe liver damage), TNM (evaluates the size and spread of cancer where *T* indicates the tumor size, *N* indicates cancer spread to near lymph nodes, and *M* indicates metastasis), the Okuda System (higher stages were correlated with a poorer prognosis), and the Cancer of the Liver Italian Program (CLIP) System (that gives a score (0, 1, or 2) to each of the following factors: a) Child-Pugh stage, b) the nodules number within the tumor and whether the tumor spreads through ≤ 50% or > 50% of the liver, c) AFP, and d) portal vein thrombosis).

#### Diagnosis

In non-cirrhotic patients, the diagnosis of HCC can be confirmed via biopsy. Patients with cirrhosis and nodules larger than 1 cm in diameter, and without typical HCC features on a first dynamic imaging examination, may undergo another imaging modality or nodule biopsy for diagnostic clarification.

#### Ultrasonography

Standard ultrasonography can detect space-occupying lesions in the liver, differentiate between cystic and solid lesions, and identify other metastatic lesions in the liver or abdomen. Contrast ultrasonography describes the hemodynamic changes in the liver tumor and can assist in the diagnosis and differentiation of liver malignancies [9].

#### Computed Tomography

Computed tomography (CT) enables precise anatomical localization of liver tumors and can be employed in assessing response to treatment. It is a highly accessible imaging modality for both primary disease but also as a staging scan for regional and distant metastatic spread.

#### Magnetic Resonance Imaging

Plain and contrast enhanced MRI are widely used to detect and diagnose liver cancer and to measure the response of liver cancer to treatment. MRI has many advantages such as multidirectional imaging, absence of radiation, high tissue resolution, and morphology-combining features including diffusion-weighted imaging, perfusion-weighted imaging, and spectrum analysis [10]. When combined with hepatocyte-specific contrast agents, better detection and diagnostic rate was observed for liver cancers ≤ 1.0 cm in size [11]. However, MRI and CT usage in the diagnosis of liver cancer still requires the incorporation of other imaging modalities, especially in other sequences of MRI (e.g., pseudocapsule) for comprehensive assessment and improving diagnostic accuracy [12].

#### Liver Biopsy

Liver biopsy offers a pathological diagnosis tool for space-occupying lesions without typical imaging of liver cancer. Liver biopsy should be performed with radiologic guidance. While liver biopsy still has a role in selected cases, its routine use in patients who meet well-defined imaging-based criteria for HCC diagnosis has become less common due to the high accuracy of modern imaging techniques. In current clinical practice, liver biopsy is generally reserved for specific scenarios where imaging findings are inconclusive or discordant, or when histological confirmation is necessary for treatment decisions. These scenarios may include cases where the imaging features are atypical, when there is a need to differentiate HCC from other liver tumors, or in cases where a biopsy may provide additional prognostic information. The histological samples are best obtained by a 16- or 18-gauge core needle puncture and cytological diagnosis by fine-needle aspiration. Bleeding and needle tract implantation are the main risks of liver biopsy. Due to the thrombosis and hemorrhagic risk in patients with chronic liver disease, liver puncture biopsy is contraindicated in this patient population. This risk is predominantly due to associated co-morbid disease (heart or kidney failure) and synthetic dysfunction of procoagulant factors in the liver, thereby impairing hemostatic function. Further contributions to thrombotic and hemorrhagic risk include nutritional deficiencies leading to endothelial dysfunction, metabolic compromise, and platelet sequestration. Therefore, given the risks of hemorrhage in this investigation, a full coagulation profile should be tested pre-procedurally in addition to a thorough family history for coagulopathies. The pathological diagnosis by liver puncture gives approximately 33% false negative results [13].

##### LI-RADS

Liver Imaging Reporting and Data System (LI-RADS) is an integrated imaging algorithm utilized for the evaluation of abnormal liver lesions, specifically in patients with cirrhosis. It has been incorporated into the 2018 HCC practice guidance by the American Association for the Study of Liver Diseases (AASLD). LI-RADS provides four distinct imaging algorithms: CT/MRI LI-RADS and contrast-enhanced ultrasound (CEUS) LI-RADS that are utilized for the imaging-based diagnosis of HCC [14]. The arterial phase plays a crucial role in the imaging-based diagnosis of HCC as the hepatic artery is the primary blood supply to HCC lesions. Optimizing the imaging protocol to capture the characteristic arterial phase enhancement is essential for the radiological diagnosis of HCC. Advancements in imaging technology, such as dual-source/dual-energy and 32-channel MRI, have enabled the detection of even smaller lesions, including those below 1.0 cm, particularly in the arterial phase. For lesions larger than 1.0 cm, in addition to arterial phase imaging, the presence of portal-delayed phase washout is characteristic of HCC. Performing multiple sequences of the arterial phase is recommended to enhance sensitivity, especially for smaller lesions with robust neovascularization, overcoming variations in blood flow kinetics and tumor characteristics. It is important to note that some larger lesions may exhibit hypovascularity in the arterial phase and demonstrate heterogeneous delayed enhancement [15].

The term “rapid washout” refers to the hypodensity or hypointensity of the lesion compared to the surrounding liver parenchyma in the portal venous or delayed phases. This washout phenomenon has a high specificity (95–96%) for the diagnosis of HCC [16]. However, the absence of washout does not exclude the possibility of HCC, as some lesions may appear hyperintense or isointense during the portal venous phase [17].

### Treatment Options

#### Liver Transplantation

Liver transplantation is considered the standard of care when aiming for curative therapy for liver cancer, particularly in patients with decompensated cirrhosis and small liver carcinoma. Liver transplantation offers the potential for complete tumor removal while addressing the underlying liver disease, providing patients with an opportunity for long-term survival and improved quality of life. Liver transplantation can be performed using organs from both deceased and living donors. The availability and preference for deceased or living donor liver transplantation may vary between countries and regions. The Milan criteria and the University of California San Francisco (UCSF) criteria are internationally recognized guidelines commonly used to determine the suitability of liver transplantation for liver cancer patients. These criteria assess the tumor burden and the overall condition of the patient to ensure optimal outcomes after transplantation [18].

#### Liver Resection

Liver resection is considered an effective treatment option for patients with HCC at stages Ia, Ib, and IIa, especially in those with good liver function reserve. Recent studies have demonstrated improved long-term outcomes following surgical liver resection [19, 20] (evidence level 1). However, it is important to note that for patients with smaller tumors (diameter ≤ 3 cm), radiofrequency ablation (RFA) has also shown comparable effectiveness as a treatment modality [21] (evidence level 1).

#### Local Ablation Therapy

Local ablation can eradicate tumor tissues through physical or chemical techniques under the guidance of imaging tools such as US, CT, and MRI. These techniques include RFA, microwave ablation, high-power focused US ablation, cryotherapy, and percutaneous ethanol injection (PEI). Ablation can be performed via percutaneous, laparoscopic, or open surgical approaches. Most small hepatic malignancies are percutaneously ablated, preferred due to lower cost and an easier and less invasive technique. Laparoscopic or open approaches are considered for subcapsular tumors, especially tumors protruding beyond the liver capsule or for liver tumors where imaging guidance is not possible. Local ablation treatment is indicated for single tumors with < 5-cm diameter or < 3 nodules in a large tumor ≤ 3 cm without distal metastasis and achieves comparable outcomes to radical resection in patients with Child–Pugh class A or B (evidence level 1) [22, 23]. Furthermore, local ablative treatment is often combined with trans-arterial chemoembolization (TACE) for patients with inoperative single or multiple tumors with a diameter of 3 to 7 cm (evidence level 1) [24].

#### Prevention

Strategies for HCC prevention include vaccination against HBV, early treatment of viral hepatitis, and long-term antiviral therapy for patients with hepatitis B-related end-stage liver cirrhosis. Vaccination against HBV is a crucial preventive measure, particularly in regions where mother-to-child transmission is a significant contributor to HBV-associated HCC. Additionally, early treatment of viral hepatitis, especially in the context of HBV, plays a vital role in reducing the risk of liver cirrhosis development and subsequent HCC [25].

#### Surveillance and Follow-Up

Surveillance for HCC is recommended for high-risk populations and compensated cirrhotic patients to achieve improved clinical outcomes. Additionally, patients with non-cirrhotic HBV at high risk for developing HCC, and those with chronic liver disease and advanced fibrosis should also be included in the surveillance program. Primarily, this program is composed of surveillance using ultrasound, which should be implemented in all cirrhotic patients every 6 months regardless of the etiology of cirrhosis.

#### Data Extraction

The data that was extracted included the guideline name, country, publication year, recommendation (yes, no, or not reported) regarding risk factors, staging, diagnosis, management, treatment options, prevention, surveillance, and follow-up of HCC and the risk of bias. Two reviewers completed data extraction for each included article and verified the findings with the larger research team.

#### Quality Assessment

The risk of bias was assessed at the individual study level using the Appraisal of Guidelines Research and Evaluation (AGREE) II tool [26]. The AGREE-II tool assesses CPGs through six domains using 23 questions on a 7-point scale. Review authors were required to provide a score per domain for a total of six quality scores. The quality of evidence was evaluated by two reviewers and then also confirmed by the whole research team. The score for each domain, ranging between 0 and 100%, was calculated via the formula: (actual score − minimal possible score)/ (maximal possible score − minimal possible score) × 100 to achieve a % [26]. A guideline is ‘strongly recommended’ if majority of the domains (> five) are scored above 60% [26].

## Results

### Description of Studies

**Fig. 1:**
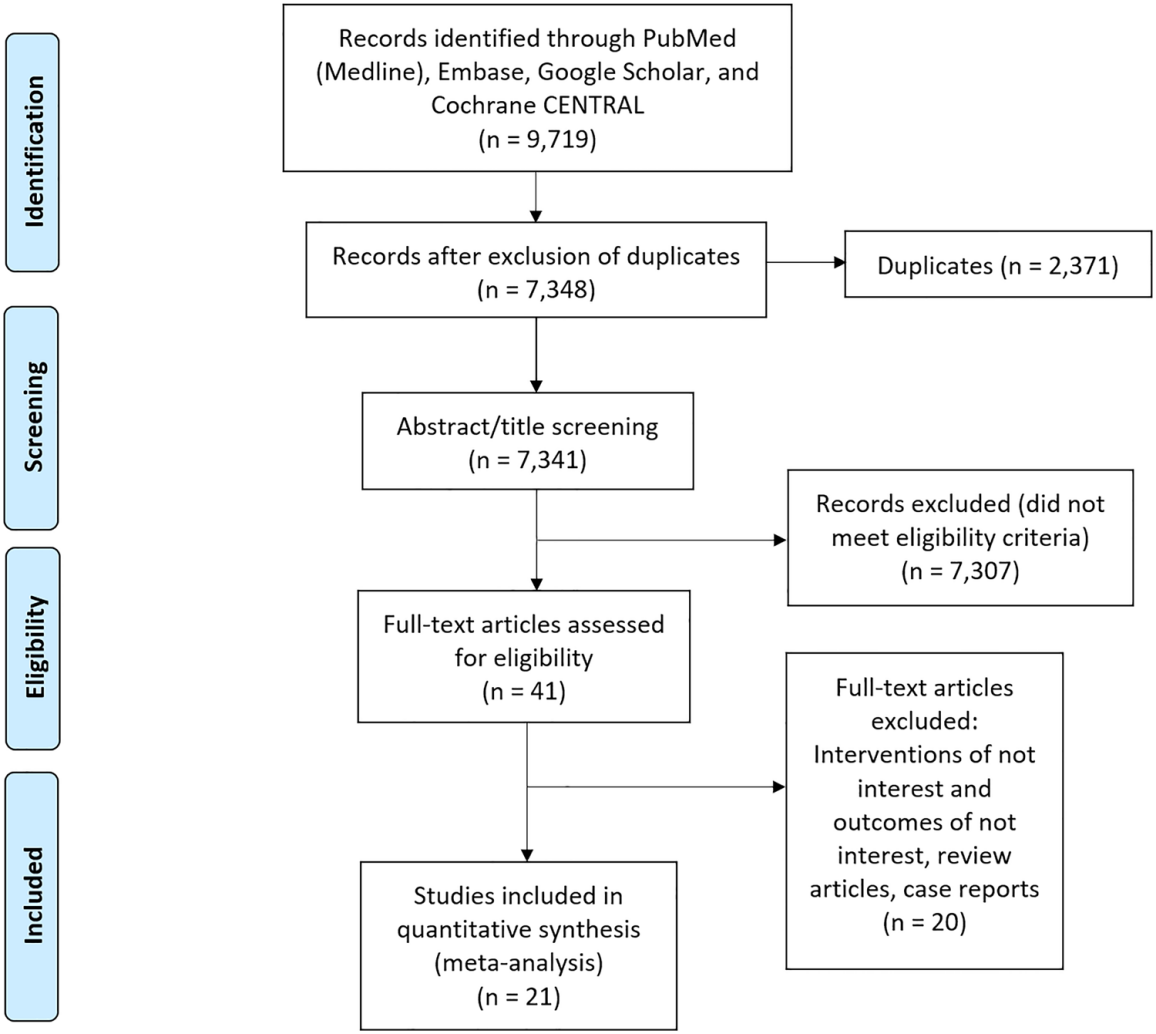
The PRISMA flow diagram of study selection

The search strategy resulted in 9719 records from the databases. Duplicates (*n* = 2371) were subsequently removed, and 7348 titles and abstracts were screened. A total of 41 articles were chosen for full-text review and 21 met the inclusion criteria [27–47]. The PRISMA checklist of study selection is outlined in Fig. 1. The included articles involved HCC guidelines from 16 different regions in the world. The year of publication ranged from 2004 to 2022. The summary of included guidelines is reported in Table 1.

**Table 1:** Summary of included studies

Guideline title	Study	Year of publication	Region
Australian recommendations for the management of hepatocellular carcinoma: a consensus statement	Lubel et al. [29]	2021	Australia
Brazilian Society of Hepatology updated recommendations for diagnosis and treatment of hepatocellular carcinoma	Chagas et al. [28]	2020	Brazil
NCCN Clinical Practice Guidelines in Oncology (NCCN Guidelines^®^)	Snyder and Vauthey [30]	2020	USA
Argentinian clinical practice guideline for surveillance, diagnosis, staging and treatment of hepatocellular carcinoma	Pinero et al. [31]	2020	Argentina
Clinical practice guidelines for hepatocellular carcinoma: The Japan Society of Hepatology 2017 (4th JSH-HCC guidelines) 2019 update	Kokudo et al. [32]	2019	Japan
2018 Korean Liver Cancer Association–National Cancer Center Korea Practice Guidelines for the Management of Hepatocellular Carcinoma	Park et al. [33]	2019	Korea
2019 Update of Indian National Association for Study of the Liver Consensus on Prevention, Diagnosis, and Management of Hepatocellular Carcinoma in India: The Puri II Recommendations	Kumar et al. [34]	2019	India
Hepatocellular carcinoma: ESMO Clinical Practice Guidelines for diagnosis, treatment, and follow-up	Vogel et al. [35]	2019	Europe
EASL Clinical Practice Guidelines: Management of hepatocellular carcinoma	Galle et al. [27]	2018	Europe
Diagnosis, Staging, and Management of Hepatocellular Carcinoma: 2018 Practice Guidance by the American Association for the Study of Liver Diseases	Marrero et al. [36]	2018	USA
Barcelona Clinic Liver Cancer	Forner et al. [37]	2018	Spain
Management consensus guideline for hepatocellular carcinoma: 2016 updated by the Taiwan Liver Cancer Association and the Gastroenterological Society of Taiwan	Lu et al. [47]	2017	Taiwan
Asia–Pacific clinical practice guidelines on the management of hepatocellular carcinoma: a 2017 update	Omata et al. [38]	2017	Asia
National Cancer Centre Singapore Consensus Guidelines for Hepatocellular Carcinoma	Chow et al. [39]	2016	Singapore
Clinical guideline SEOM: hepatocellular carcinoma	Sastre et al. [40]	2015	Spain
Mexican consensus on the diagnosis and management of hepatocellular carcinoma	Sánchez-Ávila et al. [41]	2014	Mexico
Hepatocellular carcinoma: Dutch guideline for surveillance, diagnosis and therapy	Eskens et al. [42]	2014	Netherlands
ACG Clinical Guideline: The Diagnosis and Management of Focal Liver Lesions	Marrero et al. [43]	2014	USA
Latin American Association for the Study of the Liver (LAASL)	Méndez-Sánchez et al. [44]	2014	Latin America
Saudi Guidelines for the Diagnosis and Management of Hepatocellular Carcinoma: Technical Review and Practice Guidelines	Abdo et al. [45]	2012	Saudi Arabia
Multidisciplinary Canadian consensus recommendations for the management and treatment of hepatocellular carcinoma	Sherman et al. [46]	2011	Canada

### Outcomes

#### Risk Factors

The results of included outcomes were summarized in Table 2. The development of cirrhosis is a major risk factor for the development of HCC regardless of the underlying cause. All included guidelines (*n* = 21, 100%) recommended evaluating cirrhosis, HBV, and HCV as potential risk factors of HCC. Nineteen guidelines (91%) recommended evaluating obesity/NAFLD patients, due to its associated risk of HCC. Monitoring of alcohol consumption was recommended in 17 guidelines (81%), metabolic disorders in 16 CPGs (76%), Aflatoxin B1 in 12 CPGs (57%), hereditary hemochromatosis in 10 CPGs (48%), and Wilson disease in eight CPGs (38%).

**Table 2:** Summary results of included outcomes

Characteristic	Number of guidelines that recommended evaluating the characteristic (*N* = 21)	(%)^a^	(Valid %)^b^
**Risk factors**			
Cirrhosis	21	100%	100%
Hepatitis B	21	100%	100%
Hepatitis C	21	100%	100%
Alcohol	17	81%	100%
Aflatoxin B1	12	57%	100%
Obesity non-alcoholic fatty liver disease	19	91%	100%
Hereditary hemochromatosis	10	48%	100%
Wilson disease in association with cirrhosis	8	38%	100%
Type 1 glycogen storage disease	6	29%	100%
Alpha 1 antitrypsin deficiency	8	38%	100%
Metabolic disorders (i.e., obesity, diabetes, impaired glucose metabolism, metabolic syndrome, non-alcoholic fatty liver disease)	16	76%	100%
Non-alcoholic steatohepatitis	14	67%	100%
Stage IV primary biliary cirrhosis	7	33%	100%
Porphyria cutanea tarda	2	10%	100%
Older age, male sex, the severity of cirrhosis, and sustained inflammatory activity	15	71%	100%
Hepatitis D	2	10%	100%
**Staging**			
BCLC staging system	14	67%	86%
The TNM Classification System	4	19%	75%
The Okuda System	1	5%	50%
The Cancer of the Liver Italian Program System	1	5%	50%
**Diagnosis**			
In non-cirrhotic patients, the diagnosis of HCC should be confirmed by biopsy	10	48%	100%
Patients with cirrhosis and nodules larger than 1 cm in diameter and without typical HCC features on a first dynamic imaging examination (MRI or CT) may undergo another imaging modality or nodule biopsy for diagnostic clarification	21	100%	100%
Another biopsy is recommended in cases of inconclusive histology or discordant findings	3	14%	100%
**Management**			
Multidisciplinary approach	18	86%	100%
Patients with HCC who are candidates for active treatment modalities should be managed in centers where expertise is available	3	14%	100%
After resection, close follow-up is mandatory due to the high risk of liver recurrence	7	33%	100%
Patients with end-stage HCC should receive a palliative care approach by a multidisciplinary team	18	86%	100%
**Treatment options**			
Patients should be considered for liver transplantation if they satisfy the Milan criteria	20	95%	100%
Patients are optimal candidates for liver resection if they satisfy the appropriate criteria	20	95%	100%
Patients should be considered for local ablative therapies if all the appropriate criteria are satisfied	21	100%	100%
Patients should be considered for chemoembolization if they satisfy all the appropriate criteria	21	100%	100%
Radioembolization with yttrium 90-labelled glass beads is effective in inducing necrosis in HCC with a good safety profile but has not been proven to improve survival	10	48%	90%
Sorafenib is recommended in patients with the following: 1. Child A cirrhosis (preserved liver function), 2. BCLC advanced stage (metastases), and 3. not candidates for transplantation, resection, local ablative therapy	19	91%	95%
All patients with Child C cirrhosis should be offered palliative care only unless they are candidates for liver transplantation	18	83%	100%
Percutaneous ethanol injection might be recommended in cases of very early (BCLC 0) and early (BCLC A) HCC	8	38%	100%
Downstaging of HCC using loco-regional treatment for consideration of liver transplant in a suitable candidate	5	24%	100%
Stereotactic external beam radiation therapy and external beam radiotherapy as options on their own or combined with TACE for residual disease/metastatic HCC	11	52%	100%
**Prevention**			
The vaccination of all children against hepatitis B starting at birth	15	71%	100%
Vaccination of people at risk for hepatitis B infection	15	71%	100%
Post-exposure prophylaxis for hepatitis B	13	62%	100%
Post-exposure testing for hepatitis C using PCR-based test and early treatment of hepatitis C	11	52%	100%
All patients with viral hepatitis must be properly evaluated by a hepatologist for candidacy for antiviral therapy	15	71%	100%
All patients with hepatitis B-related end-stage liver cirrhosis should be considered for long term antiviral therapy	15	71%	100%
**Surveillance**			
Surveillance for HCC is recommended for high-risk populations and compensated cirrhotic patients are the main target population for screening	21	100%	100%
Patients with non-cirrhotic hepatitis B at high risk for developing HCC and those with chronic liver disease and advanced fibrosis (F3) should also be included in the surveillance program	10	48%	100%
Surveillance should be performed by abdomen ultrasound with or without AFP every six months	18	86%	100%

*HCC* hepatocellular carcinoma, *BCLC* Barcelona Clinic Liver Cancer, *AFP* alpha fetoprotein, *TACE* trans-arterial chemoembolization

^a^Percentage of included studies that included the characteristics

^b^Excluding guidelines that did not report the characteristic

#### Staging

Fourteen guidelines (67%) recommended the BCLC staging system, three (14%) recommended the TNM classification system, one (5%) recommended the Okuda System, and one (5%) used the CLIP system. The TNM staging system has demonstrated its efficacy in predicting prognosis for patients with HBV-predominant or non-HBV HCC who undergo curative resection, except among Japanese patients. For those treated with TACE, Child-Pugh or CLIP scores offer better prognostic value than other staging systems. The widely used BCLC staging system is endorsed by major liver disease associations in the Western hemisphere. However, its performance varies across populations, with conflicting results in studies involving different ethnic groups. In contrast, the Hong Kong Liver Cancer (HKLC) classification demonstrated improved predictive ability for Chinese patients with HBV-predominant HCC with intermediate or advanced disease compared to the BCLC system. Nevertheless, controversy remains in refining and implementing a universal staging system, leading to variations in treatment approaches and complicating regional and multicenter clinical trial designs.

#### Diagnosis

All guidelines (100%) recommended that patients with cirrhosis and nodules > 1 cm in diameter and without typical HCC features on a first dynamic imaging examination (MRI or CT) may undergo another imaging modality or nodule biopsy for diagnostic clarification. The diagnosis of HCC confirmed by biopsy was recommended in 10 guidelines (48%). Another biopsy is recommended by three guidelines (15%) in cases of inconclusive histology or discordant findings.

#### Management

Eighteen guidelines (86%) recommended a multidisciplinary approach (hepatologists, liver surgeons, transplant surgeons, oncologists, diagnostic radiologists, interventional radiologists, palliative care physicians, pathologists, nurses, patient education specialists, and pharmacists). Eighteen guidelines (86%) recommended that patients with end-stage HCC should receive input from palliative care physicians. Seven guidelines (33%) recommended that post-surgical resection, close follow-up is mandatory due to the high risk of liver recurrence (high level of evidence; strong recommendation). Three guidelines (14%) recommended that patients with HCC who are candidates for active treatment modalities should be ideally managed in centers where expertise is available (tertiary or quaternary level hospitals).

#### Treatment Options

Twenty guidelines (95%) recommended liver transplantation if the patients satisfy all the Milan criteria (1. a single lesion less than 5 cm or less than three lesions smaller than 3 cm each, 2. no evidence of vascular invasion or extrahepatic spread, and 3. no contraindications for liver transplantation). Additionally, 20 guidelines (95%) recommended liver resection if the following criteria exist: no cirrhosis or early cirrhosis with normal bilirubin and no clinical signs of portal hypertension, no major vascular invasion or extrahepatic spread, and the tumor is respectable. Furthermore, all included guidelines endorsed local ablative therapies if all aspects of the following criteria were satisfied (Grade A): 1. Child A or B cirrhosis patients, 2. lesions smaller than 4 cm in diameter, and 3. no extrahepatic spread. All guidelines (100%) recommended that all patients should have chemoembolization in cases where the following criteria were completed (Grade A): 1. multifocal unresectable lesions, 2. compensated Child HAV or HBV cirrhosis with bilirubin level < 50 mmol/L, 3. patent portal vein, and 4. no vascular invasion or extrahepatic spread. Ten guidelines (48%) recommended that radioembolization may be offered to patients with the multifocal unresectable disease and Child A or B cirrhosis who have either failed TACE or have portal vein thrombosis preventing TACE and have failed sorafenib therapy (Grade D). Three guidelines (15%) recommended that radioembolization with yttrium 90-labeled glass beads is effective in inducing necrosis in HCC with a good safety profile but has not been proven to improve overall survival (Grade B). Treatment with sorafenib (multi-kinase inhibitor) is recommended in nineteen guidelines (91%) for patients who satisfy the following criteria completely (Grade A): 1. Child A cirrhosis with preserved liver function, 2. BCLC advanced stage with metastases, 3. Not candidates for transplantation, resection, local ablative therapy, or TACE. PEI is recommended in eight guidelines (38%) in cases of very early and early HCC (BCLC 0 and BCLC A, respectively) when RFA is unavailable or not technically possible, particularly in cancers smaller than 2 cm. However, in lesions > 2 cm, PEI should be discouraged due to the therapy’s association with high rates of incomplete response and local recurrence. Eleven guidelines recommended stereotactic external beam radiation therapy and external beam radiotherapy as options on their own or combined with TACE for residual disease/metastatic HCC. Five guidelines recommended down-staging of HCC using loco-regional treatment for consideration of liver transplant in a suitable candidate.

Surgeons in Eastern countries generally adopt a more aggressive approach towards HCC compared to their Western counterparts, both in terms of surgical resection and liver transplantation. In Asia, surgical resection is frequently performed for BCLC stage B and C HCC when technically feasible, contrary to recommendations by AASLD and EASL guidelines. The only definite contraindications for resection are distant metastasis, main portal vein thrombosis, and inferior vena cava thrombosis.

#### Prevention

Fifteen guidelines (71%) recommended vaccination of all children against HBV starting at birth (Grade B) and vaccination of people at greater risk for hepatitis B infection (Grade B). Post-exposure prophylaxis for hepatitis B is recommended in 13 guidelines (62%) (Grade B).

#### Surveillance and Follow-Up

Eighteen guidelines (86%) recommended that surveillance using ultrasound should be implemented in all cirrhotic patients every 6 months regardless of the cause of cirrhosis (Grade A). Six guidelines (30%) reported that surveillance of all patients with chronic HBV without evidence of cirrhosis cannot be recommended at this time but may be offered in certain high-risk groups such as patients above 40 years of age, patients with a family history of HCC, patients with high viral load, and patients with indications of advanced fibrosis by non-invasive fibrosis markers or biopsy. All included guidelines (100%) recommended that any patient with a positive ultrasound should undergo further imaging with a triphasic or four-phasic CT scan or an MRI (Grades B and C). Universal HBV immunization was recommended in 13 guidelines (62%) (high level of evidence; strong recommendation). Effective antiviral therapy, given as early as possible, is recommended in 14 guidelines (67%) for patients with HCV infection and, where indicated, for patients with chronic HBV infection (high level of evidence; strong recommendation).

#### Quality Assessment Results

The results of AGREE-II scores of all included CPGs are demonstrated in Table 3. Overall, the quality scores of CPGs varied considerably both within and across the six domains. The mean overall assessment AGREE II score was 90% indicating that all guidelines included in this study were highly recommended in the majority of domains. Most CPGs reported goals and particular populations to whom the guideline is recommended. Most target users of the guidelines were identified to be either involved or professionally related individuals. Multiple CPGs achieved a high score for clarification of key recommendations, rigor of developments, and applicability of recommendations to clinical practice. Systematic methods were not applied during the development of several CPGs, rather recommendations were established chiefly based on consensus and expert opinion. Limitations for each CPG were clearly stated in each of the articles.

**Table 3:** Standardized appraisal of Guidelines Research and Evaluation II domain scores (%)

Guideline	Author	1. Scope and purpose	2. Stakeholder involvement	3. Rigor of development	4. Clarity of presentation	5. Applicability	6 Editorial independence	Overall assessment
Australian	Lubel et al. [29]	100	96	100	100	98	100	99
AASLD	Marrero et al. [36]	100	94	100	100	99	100	99
American College of Gastroenterology (ACG)	Marrero et al. [36]	100	100	97	100	99	100	99
Argentinian Association for the Study of Liver Diseases	Pinero et al. [31]	95	80	75	95	78	80	84
Asia–Pacific	Omata et al. [38]	94	85	85	92	86	82	87
Barcelona Clinic Liver Cancer	Forner et al. [13]	96	90	79	94	76	86	87
Brazilian Society of Hepatology	Chagas et al. [28]	95	80	84	90	85	77	85
Canada	Sherman et al. [46]	99	95	90	100		97	96
Dutch	Eskens et al. [42]	94	96	85	97	76	83	88
EASL	Galle et al. [27]	100	100	99	100	99	100	100
ESMO	Vogel et al. [35]	100	100	100	100	99	100	100
Indian National Association for Study of the Liver	Kumar et al. [34]	94	78	78	92	65	86	82
Japan Society of Hepatology	Kokudo et al. [32]	98	80	91	83	96	92	90
Korean Liver Cancer Association	Park et al. [33]	96	95	93	96	91	98	95
LAASL	Méndez-Sánchez et al. [44]	99	96	76	83	89	90	89
Mexican	Sánchez-Ávila et al. [41]	88	86	73	68	90	82	81
NCCN	Snyder and Vauthey [30]	80	95	87	82	83	90	86
Saudi Arabia	Abdo et al. [45]	90	91	79	76	95	97	87
Singapore	Chow et al. [39]	85	97	83	82	97	99	88
Spanish Society of Medical Oncology (SEOM)	Sastre et al. [40]	96	86	82	90	96	93	90
Taiwan Liver Cancer Association	Lu et al. [47]	94	92	76	95	91	95	90
Mean		95	91	85	90	88	91	90

*AASLD* The American Association for the Study of Liver Diseases, *EASL* The European Association for the Study of the Liver, *ESMO* The European Society for Medical Oncology, *LAASL* Latin American Association for the Study of the Liver, *NCCN* National Comprehensive Cancer Network

## Discussion

The current review identified 21 CPGs for the assessment of HCC diagnosis and management in 16 regions worldwide. All studies included guidelines (*n* = 21, 100%) that aimed at preventing factors of cirrhosis, HBV, and HCV due to their potential risk factor towards HCC. The BCLC staging system was endorsed by a majority of studies (*n* = 14, 67%).

Hepatocellular carcinoma (HCC) exhibits considerable variation in its etiology across different regions of the world. The diverse etiological factors associated with HCC are crucial to consider when designing effective screening practices and evaluating their cost-effectiveness. In various high-income countries, chronic hepatitis B and C infections, as well as alcohol-related liver disease, remain prominent etiological factors for HCC. Conversely, in regions with a high prevalence of chronic hepatitis B, such as parts of Asia and sub-Saharan Africa, viral infection plays a more significant role. Additionally, the emergence of non-alcoholic fatty liver disease (NAFLD) as a leading cause of HCC in Western countries further highlights the evolving global landscape. Other contributing factors, such as aflatoxin exposure in certain regions of Africa and Asia, as well as hereditary conditions like hemochromatosis, Wilson’s disease, and alpha-1 antitrypsin deficiency, add to the complexity of HCC etiology worldwide.

Across 18 international guidelines for diagnosis and treatment of HCC, it was recommended that a multi-disciplinary approach be adopted, consisting of surgeons (liver and transplant), hepatologists, oncologists, radiologists (diagnostic and interventional), palliative care physicians, pathologists, and other allied health (pharmacists, nurses, and patient education specialists). Internationally, the multidisciplinary approach is considered standard care in oncology [29]. This model has been demonstrated to improve the accuracy of staging and diagnosis, deliver higher treatment rates, better patient outcomes, reduce unnecessary duration of treatment, and provide greater adherence to clinical recommendations [28]. Moreover, previous observational studies reported increased overall survival rates of HCC patients who were managed in a multidisciplinary setting [30].

For HCC surveillance by imaging, liver ultrasonography is considered the primary method. The sensitivity of ultrasonography used for HCC surveillance ranges from 60 to 90%, with a specificity of greater than 90%. CT and MRI are not appropriate routine surveillance tools for HCC imaging. The ideal HCC surveillance interval needs to match a tumor doubling time of 4 to 8 months. HCC surveillance by US every 6 months is therefore cost-effective and can increase overall survival rates [31]. Other imaging techniques, including CT, MRI, and PET, play a crucial role in identifying HCC lesions, assessing tumor size, vascular invasion, and distant metastasis [12]. Liver nodule biopsy, although controversial, remains an important diagnostic tool in HCC. It allows for histopathological examination of tumor tissue, aiding in definitive diagnosis and providing valuable prognostic information. However, biopsy is not without limitations, as it can be prone to sampling error and invasiveness and carries potential risks associated with the procedure [13]. In recent years, there has been a growing interest in non-invasive diagnostic techniques for HCC. Liquid biopsy, which involves the analysis of circulating tumor DNA and other biomarkers in the blood, holds promise for early detection and monitoring of HCC [48]. Biomarker-based approaches, such as AFP and imaging agents targeting specific molecular pathways, also show potential in improving diagnostic accuracy. These emerging non-invasive diagnostic techniques offer the advantage of reduced invasiveness and may complement or even replace traditional biopsy methods in the future, revolutionizing the field of HCC diagnosis [49].

The BCLC staging system is used and recommended by various guidelines for staging [32–34]. The BCLC covers three clinical characteristics: liver function, tumor biology, and performance status, in addition to providing treatment recommendations according to the tumor stage. The BCLC system is the most commonly adopted approach in multidisciplinary teams, due to physician acceptability and ease of use [35]. The TNM Classification System focuses on tumor characteristics and is widely accepted for its global applicability [50]. The Okuda System provides a simpler approach, considering tumor size and presence of ascites and/or bilirubin levels, but it may lack sensitivity in detecting early-stage HCC [51]. The CLIP system incorporates tumor burden, performance status, and presence of portal vein thrombosis, serving as an effective prognostic indicator. While these staging systems have demonstrated their prognostic value and practical applicability, they also possess certain limitations, such as potential subjectivity in assigning performance status and limited consideration of tumor biology [51].

In terms of HCC surgical treatment, macroscopic hepatic resection is recommended for HCC patients in whom the cancer is limited to the liver and can be excised, while the remaining liver part should be sufficient in terms of both quantity and quality to sustain life. Evaluating patients for potential liver resection for HCC include patient functional status, hepatic functional status, the degree of portal hypertension, and HCC features [33]. Image-guided percutaneous ablation is an effective treatment for ‘very early’ and ‘early’ HCC. RFA has now replaced PEI as the most common ablative method. RFA is favored over ethanol injection, demonstrating improved loco-regional control of disease and survival, and fewer treatment sessions necessary to complete treatment [36]. Liver transplantation is regarded as one of the best treatments for early-stage HCC patients, due to simultaneously treating the tumor and the underlying liver disease (which is the main risk factor for the development of new tumors). HCC arises mostly from a cirrhotic liver, where there is impaired hepatic function. Therefore, liver transplantation is regarded as definitive therapy for cirrhotic patients with HCC, due to tumor being removed with the largest possible margin and its subsequent replacement with a non-cirrhotic liver [37].

The San Francisco criteria play a significant role in determining eligibility for liver transplantation, providing guidelines for selecting patients based on tumor size, number of lesions, and vascular invasion [52]. Downstaging is a treatment strategy that offers hope for patients with initially unresectable tumors. By successfully reducing tumor size, selected patients may become eligible for curative therapies. Downstaging approaches can include locoregional therapies, systemic therapies, or a combination of both. The rationale behind downstaging lies in the potential for improving outcomes by converting initially unresectable tumors into surgically resectable ones or facilitating other curative treatment options. Patient selection criteria for downstaging typically involve tumor burden, liver function, performance status, and the likelihood of achieving successful tumor reduction. Evaluating the outcomes associated with downstaging is crucial in assessing its effectiveness in improving overall survival and disease-free survival rates [53].

### Strengths and Limitations

To the best of our knowledge, this is the first systematic critical appraisal of the published CPGs for the management of HCC. Most included CPGs in this systematic review achieved high quality ratings regarding methodological rigor across multiple AGREE-II domains.

We acknowledge the limitations of the selected time frame and the potential impact of recent developments on HCC management. We emphasize the need for future updates and revisions of clinical practice guidelines to incorporate the latest evidence and advancements in epidemiology, imaging, and treatment modalities. Furthermore, there have been advancements that have occurred during this time period, including the introduction of DAA treatments for hepatitis C, new imaging techniques, and the emergence of lenvatinib and immunotherapy. One important limitation of this study is the inherent challenge of including CPGs from under-resourced settings in the evaluation of global HCC management. It is well recognized that HCC is a significant health burden in these regions, where limited healthcare resources may restrict the development and implementation of comprehensive CPGs.

Another limitation was that most included recommendations had low-quality research evidence or expert consensus only. Although the AGREE II instrument has well-known validity and reliability for assessing CPG development processes, it does not appraise clinical content and strength of evidence of recommendations extracted from the guidelines. However, this limitation is common to all present critical appraisal tools.

### Clinical Implications

The high incidence of HCC in developed countries suggests inadequate management of liver disease and inadequate tumor surveillance programs. It is crucial to implement cost-effective methods for stratifying patients based on HCC severity and adapting surveillance approaches accordingly. Chemo-preventive strategies for HCC are recommended, but molecular findings have not yet improved prognostic assessment or therapeutic decision-making. Understanding the association between molecular subclasses and patient responses is necessary for personalized treatment approaches. Next-generation sequencing technologies can aid in identifying genetic alterations, classifying tumor composition, and understanding the tumor microenvironment, enabling the development of prognostic biomarkers for clinical use. Utilizing tumor markers from the tissue, blood, and urine can assist in early diagnosis, surveillance, and treatment response prediction for high-risk patients. These markers also provide insights into treatment resistance mechanisms and can aid in stratifying patients for appropriate adjuvant and palliative therapies.

Our work contributes significantly to clinical decision-making in HCC by offering unique insights and perspectives. Through a critical analysis of existing CPGs for HCC, we add value by synthesizing global practices and identifying commonalities or divergences across different regions. This approach provides a comprehensive overview that goes beyond individual CPGs, taking into account demographic and resource variations in the broader landscape of HCC management. We emphasize the importance of evidence-based medicine and highlight the potential for our study to inform future updates or revisions of CPGs. By incorporating the evolving understanding of HCC, our findings can contribute to the continuous refinement of CPGs, ensuring they reflect the most up-to-date knowledge and improve patient outcomes.

## Conclusion

The majority of CPGs recommended evaluating cirrhosis, hepatitis B, and hepatitis C as potential risk factors for HCC. The BCLC staging system was endorsed by a significant number of guidelines. Multidisciplinary approaches involving various healthcare professionals were recommended internationally, leading to improved staging accuracy, treatment rates, patient outcomes, and adherence to clinical recommendations. Liver ultrasonography every 6 months was deemed cost-effective and effective for HCC surveillance. Surgical options such as hepatic resection, image-guided percutaneous ablation, and liver transplantation were recommended based on patient characteristics and disease stage. Ongoing evidence accumulation necessitates continuous re-evaluation and updating of recommendations. We recommend focusing on the following objectives include implementing public health policies, developing effective screening methods, and exploring molecular subclasses in clinical trials for improved treatment outcomes.

## Data Availability

Data are available upon reasonable request from correspond author.

## References

[1] Bosetti C, Turati F, La Vecchia C (2014). Hepatocellular carcinoma epidemiology. Best Pract Res Clin Gastroenterol.

[2] Bray F (2018). Global cancer statistics 2018: GLOBOCAN estimates of incidence and mortality worldwide for 36 cancers in 185 countries. CA Can J Clin.

[3] Bosch FX, Ribes J, Borràs J. Epidemiology of primary liver cancer. In: Seminars in liver disease. ^©^ 1999 by Thieme Medical Publishers, Inc.; 1999. 10.1055/s-2007-1007117 10518307

[4] Chen LT (2020). Pan-Asian adapted ESMO Clinical Practice Guidelines for the management of patients with intermediate and advanced/relapsed hepatocellular carcinoma: A TOS–ESMO initiative endorsed by CSCO, ISMPO, JSMO, KSMO MOS and SSO. Ann Oncol.

[5] Chidambaranathan-Reghupaty S, Fisher PB, Sarkar D (2021). Hepatocellular carcinoma (HCC): Epidemiology, etiology and molecular classification. Adv Cancer Res.

[6] McGlynn KA, London WT (2011). The global epidemiology of hepatocellular carcinoma: Present and future. Clin Liver Dis.

[7] El-Serag HB (2016). Risk of hepatocellular carcinoma after sustained virological response in veterans with hepatitis C virus infection. Hepatology.

[8] Page MJ, et al. The PRISMA 2020 statement: An updated guideline for reporting systematic reviews. Int J Surg. 2021;88. 10.1016/j.ijsu.2021.105906 33789826

[9] Salvatore V (2016). Imaging diagnosis of hepatocellular carcinoma: Recent advances of contrast-enhanced ultrasonography with SonoVue®. Liver Cancer.

[10] Lee YJ (2015). Hepatocellular carcinoma: Diagnostic performance of multidetector CT and MR imaging—a systematic review and meta-analysis. Radiology.

[11] Joo I, Lee JM (2016). Recent advances in the imaging diagnosis of hepatocellular carcinoma: Value of gadoxetic acid-enhanced MRI. Liver Cancer.

[12] Zhou J (2018). Guidelines for diagnosis and treatment of primary liver cancer in China (2017 Edition). Liver Cancer.

[13] Forner A (2008). Diagnosis of hepatic nodules 20 mm or smaller in cirrhosis: Prospective validation of the noninvasive diagnostic criteria for hepatocellular carcinoma. Hepatology.

[14] Chernyak V (2018). Liver imaging reporting and data system (LI-RADS) version 2018: Imaging of hepatocellular carcinoma in at-risk patients. Radiology.

[15] Luca A (2010). Multidetector-row computed tomography (MDCT) for the diagnosis of hepatocellular carcinoma in cirrhotic candidates for liver transplantation: Prevalence of radiological vascular patterns and histological correlation with liver explants. Eur Radiol.

[16] Furlan A (2011). Hepatocellular carcinoma in cirrhotic patients at multidetector CT: Hepatic venous phase versus delayed phase for the detection of tumour washout. Br J Radiol.

[17] Cereser L (2010). Comparison of portal venous and delayed phases of gadolinium-enhanced magnetic resonance imaging study of cirrhotic liver for the detection of contrast washout of hypervascular hepatocellular carcinoma. J Comput Assist Tomogr.

[18] Zhang HM, et al. Milan criteria, University of California, San Francisco, criteria, and model for end-stage liver disease score as predictors of salvage liver transplantation. In: Transplantation Proceedings. Elsevier; 2015. 10.1016/j.transproceed.2014.10.046 25769587

[19] Liu PH (2016). Surgical resection versus radiofrequency ablation for single hepatocellular carcinoma ≤ 2 cm in a propensity score model. Ann Surg.

[20] Feng K (2012). A randomized controlled trial of radiofrequency ablation and surgical resection in the treatment of small hepatocellular carcinoma. J Hepatol.

[21] Chen MS, et al. A prospective randomized trial comparing percutaneous local ablative therapy and partial hepatectomy for small hepatocellular carcinoma. Ann Surg. 2006;243(3):321. 10.1097/01.sla.0000201480.65519.b8 PMC1448947 16495695

[22] Hasegawa K (2014). Comparison of the therapeutic outcomes between surgical resection and percutaneous ablation for small hepatocellular carcinoma. Ann Surg Oncol.

[23] Li L (2012). Clinical outcomes of radiofrequency ablation and surgical resection for small hepatocellular carcinoma: A meta-analysis. J Gastroenterol Hepatol.

[24] Morimoto M (2010). Midterm outcomes in patients with intermediate-sized hepatocellular carcinoma: a randomized controlled trial for determining the efficacy of radiofrequency ablation combined with transcatheter arterial chemoembolization. Cancer.

[25] Nguyen MH. Hepatitis B virus: Advances in prevention, diagnosis, and therapy. Clin Microbiol Rev. 2020;33(2). 10.1128/CMR.00046-19 PMC7048015 32102898

[26] Brouwers MC (2010). AGREE II: Advancing guideline development, reporting and evaluation in health care. CMAJ.

[27] Galle (2018). EASL clinical practice guidelines: Management of hepatocellular carcinoma. J Hepatol.

[28] Chagas AL (2020). Brazilian society of hepatology updated recommendations for diagnosis and treatment of hepatocellular carcinoma. Arq Gastroenterol.

[29] Lubel JS (2021). Australian recommendations for the management of hepatocellular carcinoma: A consensus statement. Med J Aust.

[30] Snyder RA, Vauthey J. Hepatobiliary cancers. The MD Anderson Surgical Oncology Handbook. 6th ed. 2020.

[31] Piñero F (2020). Argentinian clinical practice guideline for surveillance, diagnosis, staging and treatment of hepatocellular carcinoma. Ann Hepatol.

[32] Kokudo N (2019). Clinical practice guidelines for hepatocellular carcinoma: The Japan Society of Hepatology 2017 (4th JSH-HCC guidelines) 2019 update. Hepatol Res.

[33] Park JW (2019). 2018 Korean Liver Cancer Association-National Cancer Center Korea practice guidelines for the management of hepatocellular carcinoma. Gut Liver.

[34] Kumar A (2020). 2019 update of Indian national association for study of the liver consensus on prevention, diagnosis, and management of hepatocellular carcinoma in India: The puri II recommendations. J Clin Exp Hepatol.

[35] Vogel A (2018). Hepatocellular carcinoma: ESMO clinical practice guidelines for diagnosis, treatment and follow-up. Ann Oncol.

[36] Marrero JA (2018). Diagnosis, staging, and management of hepatocellular carcinoma: 2018 practice guidance by the American Association for the Study of Liver Diseases. Hepatology.

[37] Forner A, Reig M, Bruix J (2018). Hepatocellular carcinoma. Lancet.

[38] Omata M (2017). Asia-Pacific clinical practice guidelines on the management of hepatocellular carcinoma: A 2017 update. Hep Intl.

[39] Chow PK (2016). National cancer centre Singapore consensus guidelines for hepatocellular carcinoma. Liver Cancer.

[40] Sastre J (2015). Clinical guideline SEOM: hepatocellular carcinoma. Clin Transl Oncol.

[41] Sánchez-Ávila JF (2015). Mexican consensus on the diagnosis and management of hepatitis C infection. Ann Hepatol.

[42] Eskens F (2014). Hepatocellular carcinoma: Dutch guideline for surveillance, diagnosis and therapy. Neth J Med.

[43] Marrero JA (2014). ACG clinical guideline: The diagnosis and management of focal liver lesions. Am J Gastroenterol.

[44] Méndez-Sánchez N (2014). Latin American Association for the Study of the Liver (LAASL) clinical practice guidelines: Management of hepatocellular carcinoma. Ann Hepatol.

[45] Abdo AA (2012). Saudi guidelines for the diagnosis and management of hepatocellular carcinoma: Technical review and practice guidelines: Created and endorsed by the Saudi Association for the Study of Liver Diseases and Transplantation and the Saudi Oncology Society. Ann Saudi Med.

[46] Sherman M (2011). Multidisciplinary Canadian consensus recommendations for the management and treatment of hepatocellular carcinoma. Curr Oncol.

[47] Lu SN (2018). Management consensus guideline for hepatocellular carcinoma: 2016 updated by the Taiwan Liver Cancer Association and the Gastroenterological Society of Taiwan. J Formos Med Assoc.

[48] Bhan I, Hoshida Y (2019). Liquid biopsy in hepatocellular carcinoma. Hepatocellular carcinoma: Translational precision medicine approaches.

[49] Pan A. Circulating biomarkers for the early diagnosis and management of hepatocellular carcinoma with potential application in resource-limited settings. Diagnostics (Basel). 2023;13(4). 10.3390/diagnostics13040676 PMC9954913 36832164

[50] O'Sullivan B (2017). The TNM classification of malignant tumours-towards common understanding and reasonable expectations. Lancet Oncol.

[51] Kinoshita A (2015). Staging systems for hepatocellular carcinoma: Current status and future perspectives. World J Hepatol.

[52] Unek T (2011). Comparison of Milan and UCSF criteria for liver transplantation to treat hepatocellular carcinoma. World J Gastroenterol.

[53] Chen X, et al. Downstaging therapies for unresectable hepatocellular carcinoma prior to hepatic resection: A systematic review and meta-analysis. Front Oncol. 2021;11. 10.3389/fonc.2021.740762 PMC8639517 34868936

